# Integrating Automatic Speech Recognition Technology Into Vocabulary Learning in a Flipped English Class for Chinese College Students

**DOI:** 10.3389/fpsyg.2022.902429

**Published:** 2022-07-15

**Authors:** Michael Yi-Chao Jiang, Morris Siu-Yung Jong, Na Wu, Bin Shen, Ching-Sing Chai, Wilfred Wing-Fat Lau, Biyun Huang

**Affiliations:** ^1^Department of Curriculum and Instruction, Faculty of Education, The Chinese University of Hong Kong, Hong Kong, China; ^2^Centre for Learning Sciences and Technologies, Hong Kong Institute of Educational Research, The Chinese University of Hong Kong, Hong Kong, China; ^3^College of International Business, Shenyang Normal University, Shenyang, China; ^4^School of Foreign Languages, Fuzhou University, Fuzhou, China; ^5^Center for Foreign Language Education and Teaching, School of Foreign Languages, Fuzhou University, Fuzhou, China

**Keywords:** automatic speech recognition, flipped classroom, vocabulary learning, CAF framework, trade-off effect

## Abstract

Although the automatic speech recognition (ASR) technology is increasingly used for commercial purposes, its impact on language learning has not been extensively studied. Underpinned by the sociocultural theory, the present work examined the effects of leveraging ASR technology to support English vocabulary learning in a tertiary flipped setting. A control group and an experimental group of college students participated in a 14-week study. Both groups had their English classes in a flipped fashion, but the experimental group was assigned with ASR-assisted oral tasks for pre-class self-learning. The pre- and post-intervention in-class task performance of both groups was audio-recorded and transcribed for data analysis. The triadic complexity-accuracy-fluency (CAF) framework was adopted to evaluate the participants' vocabulary learning. The between- and within-subjects effects were examined mainly through procedures of MANCOVA and mixed-design repeated measures ANCOVA. Results showed that on all the metrics of lexical complexity and speed fluency, the experimental group outperformed the control group, and had significant growth over time. On the other hand, the control group only improved significantly overtime on the G-index. On lexical accuracy, there was no significant difference between the two groups, and the within-subjects effect was not significant for either group. The findings lent some support to Skehan's Trade-off Hypothesis and discussions were conducted regarding the triarchic CAF framework.

## Introduction

Learning a second or foreign language (L2/FL) usually requires a substantial amount of constant corrective feedback from a source other than learners' perceptions (Franco et al., 2010). Given that sounds are filtered through their mother tongue (McCrocklin, 2016), L2/FL learners are quite unlikely to monitor their own oral speech practice in the target language. In addition, the feedback provided by language teachers for each learner is oftentimes subject to time and space restraints. With the advancement of educational technology and artificial intelligence, especially in the domain of intelligent computer-assisted language learning (iCALL), the automatic speech recognition (ASR) technology is progressively regarded as a conceivable solution to address that issue (Mroz, 2018; McCrocklin, 2019; Evers and Chen, 2020; Jiang et al., 2021; Zhai et al., 2021). ASR-based technologies and applications attract L2/FL researchers' and practitioners' attention (McCrocklin, 2016; Penning de Vries et al., 2020), owing to the features such as considerable amounts of practice, consistent and unbiased feedback, and diverse forms of visual representations (Levis, 2007). According to Rassaei (2021), those features are integrally linked with and emphasized in sociocultural theory as critical characteristics of an effective classroom teaching. Moreover, apart from more opportunities for extensive interaction in the target language and real-time feedback, ASR-based technology can also provide L2/FL learners with more control over their self-learning, thus creating a less threatening self-paced environment for individual learners when learning to speak in the target language (Jiang et al., 2021).

A growing number of empirical studies examining the effect of ASR technology on L2/FL learning have been conducted in the past decade with the majority of them dedicated to research on L2/FL pronunciation (McCrocklin, 2016; Evers and Chen, 2020), some dedicated to improving oral grammatical skills and complexity (Penning de Vries et al., 2020; Jiang et al., 2021), while far less to vocabulary learning (Bashori et al., 2021), despite the paramount role of productive vocabulary learning in L2/FL learning (Schmitt, 2010; Li and Hafner, 2021). Previous research has also lent some support to the effects of iCALL technologies on vocabulary knowledge (e.g., Chen and Hsu, 2019; Soyoof et al., 2022), thus making the present attempt to integrate ASR technology into vocabulary learning in an L2/FL classroom deserve attention. Moreover, most of the studies were carried out in conventional L2/FL settings, where the use of the ASR-based technology might not be to the fullest due to the limited in-class time or less-structured pre-class self-learning (Jiang et al., 2020). Besides, those studies mainly focused on the technological integration of the ASR-based applications, but few detailed the instructional design and implementation as much. Because of such insufficient description of how the ASR-based technology was pedagogically integrated into task-based language learning in those studies, their pedagogical implications for future studies may be diminished. Furthermore, an overwhelming majority of the studies utilized self-reported data (e.g., attitudes toward the use of ASR technology) and adopted only overall measures of the students' academic performance (e.g., overall assessment of oral proficiency) to examine the effects of ASR technology. In contrast, objective and fine-grained measures such as those based on the tripartite framework of complexity, accuracy and fluency (CAF) (Skehan, 1996) are barely used. Specific measures of learners' linguistic performance are regarded as more direct gauges in depicting students' language learning, and thus may further contribute to diversifying the instructional design of the flipped classroom approach (Jiang et al., 2021). Therefore, little is known about how L2/FL learners' vocabulary learning is affected by the ASR-based technology. With those research gaps identified, the present study aimed to utilize the CAF framework to investigate how the ASR-based technology might facilitate students' L2/FL vocabulary learning. The findings may contribute to a better understanding of exploiting ASR-based applications and shed light on the course design in flipped EFL classrooms.

## Related Works

### A Sociocultural Theoretical (SCT) Perspective

In the past decades, there emerges an accumulating interest in taking a sociocultural theoretical perspective to research L2/FL learning (Rassaei, 2014, 2020). According to the sociocultural approach, language development is rooted in dialogic interactions (Ellis, 2009), and learners are empowered to perform challenging tasks which may exceed their abilities through social interaction with assistance from other capable learners or social environment and artifacts (Vygotsky, 1978). Traditionally associated with Vygotsky's (1978, 1987) work, SCT relates social interaction to individual cognitive development. Central to the notion of SCT lies the stance that higher forms of learning and thinking originate from social interaction (Vygotsky, 1978; Villamil and de Guerrero, 2006). Different from other second language acquisition (SLA) and cognitive theories such as the information-processing approach and interactionist theory, which view social interaction and information processing from such interaction as separate practices though admitting the significant role of social interaction in second language learning, SCT holds “social interaction (with both humans and artifacts participating dialogically) is internalized, the external-dialogic becomes the internal-dialogic, and a socially constructed dialogic mind emerges” (Villamil and de Guerrero, 2006, p. 24). Vygotsky's (1978) zone of proximal development (ZPD) is defined as the distance between what a learner can do with assistance and what the same learner can perform independently. Put it another way, there are thereby distinctions between a learner's actual level of language learning improvement when engaged in self-learning without external support and his/her potential level of development when facilitated by assisted and collaborative performance. From this perspective, the instant feedback on language production as generated by the ASR software could be conceptualized as the social artifact/mediator, interaction with which can lead to growth and improvement in vocabulary learning on the learners' side.

Important SCT concepts to understand and investigate the potential effects of ASR-enhanced technology on vocabulary learning in an L2/FL context include mediation, internalization, and developmental change. As Villamil and de Guerrero (2006) analyzed, the adult human mind has to firstly go through a sociocultural mediation to transform from lower forms of thinking (natural memory, basic perception) to higher forms of thinking (logical reasoning, problem solving). Mediation by others, mediation by self, and mediation by artifacts are the three forms of mediation postulated by SCT; moreover, internalization of mediation is a developmental process to achieve higher order of thinking, and Wertsch's (1979) categorization of regulation stages proposed learners move from other-regulation to self-regulation in the transition from interpsychological to intrapsychological activity. To be specific, when ASR technology is integrated into vocabulary learning, learners could receive assistance from social artifact (feedback from ASR software) and go through a sociocultural mediation by artifacts and self to transform from lower to higher forms of thinking, move from other-regulation (i.e., performing with assistance from ASR software) to self-regulation (i.e., capable of independent performance of oral task) in the transition from interpsychological to intrapsychological activity.

### ASR-Assisted Vocabulary Learning in Flipped EFL Classrooms

Owing to their easy accessibility and ubiquity, smartphones and tablets can be utilized for providing constant feedback and mediation to language learners (Rassaei, 2021), and the past decades have witnessed a research boom in the field of iCALL. In particular, ASR has emerged as one of the more promising iCALL technologies which is empowered by computer-based processes of decoding and transcribing oral language usually into text form (Kim, 2006). When ASR technology is integrated in a pedagogically sound way, it facilitates interactive learning environments (Wang and Young, 2014), offers instant assessment and feedback on language pronunciation and language use (Franco et al., 2010), enables easily accessible oral practice opportunities beyond time and space limitations (Torlakovic and Deugo, 2004), and reduces L2/FL speaking anxiety (Bashori et al., 2020). With these merits noted, ASR is considered beneficial for L2/FL oral practice. Specifically, mounting evidence has been accumulated on the effectiveness of applying ASR to enhance L2/FL pronunciation (Neri et al., 2008; McCrocklin, 2016) and new and sporadic attempts were made on improving oral grammatical skills (Penning de Vries et al., 2014, 2020). However, notably scant attention has been focused on the employment of ASR in promoting vocabulary learning in the L2/FL learning contexts (Bashori et al., 2021).

In view of the tenet of the flipped classroom approach and the Bloom's taxonomy (Anderson and Krathwohl, 2001), lower-level learning objectives (i.e., remembering- and understanding-oriented content), such as vocabulary learning in L2/FL classrooms, could be achieved through students' pre-class self-study. Flipped classroom is considered a well-matched approach for incorporating ASR-based technology in L2/FL vocabulary learning because the pre-class self-learning is usually well-organized and more self-paced than in a conventional classroom. Moreover, students in a flipped classroom are expected to spend adequate time in self-learning and practicing prior to attending class (Jong, 2017; Jong et al., 2019). Consequently, empirical studies are needed to examine how flipped classroom approach could facilitate the integration of ASR technology in the context of L2/FL learning.

Vocabulary plays a critical role in L2/FL learning given vocabulary knowledge being found to significantly predict the four essential language skills (Schmitt, 2010; Milton, 2013). But for learners of English as a second or foreign language (ESL/EFL), vocabulary acquisition often poses a challenging burden (Lo and Murphy, 2010; Webb and Nation, 2017). Moreover, L2/FL class time usually appears inadequate for vocabulary learning (Nation, 2006). In most cases, L2/FL learners may need to seek alternative resources to learn words independently out of class (Teng, 2020). Luckily, technological advancements have induced such learning opportunities with iCALL approaches, such as captioned videos (Teng, 2019, 2022), mobile games (Chen and Hsu, 2019; Abdulrahman and Jullian, 2020; Rahman and Angraeni, 2020) and virtual reality tools (Madini and Alshaikhi, 2017; Tai et al., 2020). Results have attested to the positive effects of technologies on learners' vocabulary knowledge, especially productive vocabulary learning, and their self-efficacy in vocabulary learning (e.g., Li and Hafner, 2021; Soleimani et al., 2022), which could pave the way for integrating ASR into vocabulary learning. Among the scarce endeavors, Bashori et al. (2021) conducted a quasi-experimental study with Indonesian secondary school students and reported students from the two ASR intervention groups (using two different ASR websites) outperformed the regular class group in their knowledge of the targeted vocabulary and emotional states (i.e., anxiety and enjoyment).

It is worthwhile to note in Bashori et al.'s (2021) study, vocabulary knowledge was assessed using written vocabulary test on the targeted words. This approach presented a relatively simplified assessment of the learners' mastery of the targeted vocabulary in an arbitrary fashion of correct or incorrect answers while jeopardizing an informative insight into the multi-dimensional construct of vocabulary competence. With the endorsement of the multi-componential nature of linguistic competence (Norris and Ortega, 2009), it is important for researchers to examine domain-specific outcome measures, including L2 complexity, accuracy and fluency. For example, learners' lexical and syntactic complexity in English oral performance was noticeably improved when engaged in ASR-based oral tasks for a semester (Jiang et al., 2021). Feedback generated from iCALL-based speaking practices could lead to more accurate utterances (Mackey and Goo, 2007). ASR-based pronunciation system was found to be equally capable of diagnosing human pronunciation errors as human raters did at the segmental level, and it was found that learners' varied pronunciation learning needs were met by using the ASR technology (Xiao and Park, 2021). That said, to bridge the research void, we intend to adopt the triadic CAF framework to measure the learners' vocabulary development, in the hope of retrieving a detailed diagnostic evaluation of their vocabulary learning as a result of ASR-enhanced oral practice.

### The Triadic Componential CAF Framework

Language proficiency is perceived as a multi-componential, multilayered, and multifaceted construct rather than a unitary one, and its principal components can be fruitfully captured by the framing of CAF (Housen et al., 2012; Jiang et al., 2021). Skehan (1996, 1998) theoretically combined the three constructs into one proficiency model and provided the working definitions which are still in use in areas such as SLA. Ever since, a heated debate has surrounded the issue of quantifying language learners' output in both written and spoken form.

Complexity concerns size, elaborateness, richness, and diversity of the language learners' linguistic system (Bui, 2021). In the literature pertaining to CAF, complexity is generally assessed through the competence to use a wide and varied range of advanced vocabulary and sophisticated structures in the target language (Skehan, 1998; Ellis, 2003, 2008; Housen et al., 2012). Because of its polysemous nature, complexity in language learning retains multiple meanings (Michel, 2017; Bulté and Roothooft, 2020) and is the most debated construct of the CAF triad (Pallotti, 2009). Following Michel (2017), complexity can be applied to three different dimensions, i.e., developmental, cognitive and linguistic complexity. Empirical studies converge to show that linguistic complexity is the most commonly measurable construct (Bui, 2021). Operationally, measures created for assessing linguistic complexity are dichotomized into two broad categories: lexical complexity and syntactic complexity. In the context of this study, lexical complexity is adopted as an indicator of EFL learners' vocabulary learning performance. In literature, a considerable number of EFL studies have investigated the role of lexical complexity in language learning, but most of their data were written English (e.g., Barrot and Gabinete, 2021; Han et al., 2021). In contrast, few studies have investigated oral lexical complexity in the context of EFL learning (Bulté and Roothooft, 2020). In response, the present study seeks to address this gap by examining EFL learners' lexical complexity in their oral English. Although lexical complexity can be investigated through various aspects such as diversity, density and sophistication (Skehan, 2003; Bulté and Housen, 2012), complexity is primarily shaped by lexical diversity, and operationally, lexical diversity is usually the most frequently used measure for assessing lexical complexity (e.g., Ågren et al., 2012; Han et al., 2021) and lexical development (e.g., Crossley et al., 2009). In the current study, therefore, as has been the case for most studies so far, complexity is quantified using lexical diversity in the tripartite CAF framework.

Accuracy is arguably the most straightforward and internally consistent construct of the CAF triad (Housen and Kuiken, 2009). Fundamentally, accuracy is generally defined as the degree to which a learner's language performance (and the target language system that underlies his or her performance) deviates from the native-like use (Wolfe-Quintero et al., 1998; Pallotti, 2009; Barrot and Gabinete, 2021). The deviations are typically labeled as “errors” and based on the classification of the errors, the measures of linguistic accuracy may concern lexis, morphology, syntax, phonology, and pragmatics (Chavez, 2014). In L2/FL studies, researchers showcased that accuracy could be reliably and validly measured by error-free metrics, such as calculating the number of error-free clauses of all clauses or the ratio of error-free T-units to all T-units (Polio and Shea, 2014; Barrot and Gabinete, 2021). But in China, English is learned and used as an FL rather than an L2, and for most non-English major undergraduates, the proportion of error-free utterances generated in their classroom talk is assumed to be exceedingly low. Therefore, given the potential floor effect of error-free metrics, the present study adopted error-based metrics for quantifying accuracy. According to previous studies (e.g., Liao, 2020), lexical accuracy and morphosyntactic accuracy are two most frequently adopted metrics. To be specific, lexical accuracy involves an ability to retrieve an appropriate word and use it correctly in a specific context, while morphosyntactic accuracy focuses on aspects such as agreement, inflection as well as retrieving an appropriate structure or organizing constituents in order. In the context of the present study, therefore, lexical accuracy is adopted as an accuracy indicator to measure students' vocabulary language learning, which was operationalized through students' lexical errors in their oral English.

In general usage, fluency is often understood as a language learner's overall language proficiency that particularly relates to the ease, eloquence, smoothness and native-likeness of their speech or writing (Lennon, 1990; Chambers, 1997; Van Waes and Mariëlle, 2015). A fluent L2/FL learner is believed to be capable of producing the target language with native-like rapidity, pausing, hesitation or reformulation. In contrast to complexity and accuracy, which are primarily associated with the current state of the learner's interlanguage knowledge, fluency is oftentimes a phonological phenomenon (Housen et al., 2012). Likewise, fluency is also multi-dimensional as the other two constructs in the tripartite CAF framework. Following Skehan and other researchers (Skehan, 2003, 2009; Tavakoli, 2016; Tavakoli et al., 2016), fluency can be examined through its subdimensions such as speed fluency (the rate and density of linguistic units produced), breakdown fluency (number, length, and location of pauses) and repair fluency (false starts, mis-formulations, self-corrections, and repetitions) (Housen et al., 2012). According to Lambert and Kormos (2014), fluency metrics that are conceptualized based on speech rate (i.e., a ratio of syllables produced to time taken to produce them) are the most frequently used measures. Conversely, dysfluency metrics (i.e., breakdown fluency, repair fluency) that are based on filled/unfilled pauses, hesitations, false starts, and so on did not show a strong association with learners' overall oral proficiency assessed by native speakers (Kormos and Dénes, 2004). Moreover, compared with breakdown or repair fluency, speed fluency is more linked to the L2/FL lexicon in oral output, and therefore it is posited to be a manifestation of a more advanced proficiency level pertaining to vocabulary learning. As such, in the context of the present study, speed fluency was employed as a fluency indicator to evaluate students' vocabulary learning performance.

### The Trade-Off Hypothesis in Task-Based Language Learning

Earlier known as the Limited Attentional Capacity Model, the Trade-off Hypothesis (Skehan, 2009) states that learners' attentional resources are limited, and interlocutors must allot their attentional resources a task requires during the processes (Sample and Michel, 2014). As a result, if task demands exceed the available attentional resources, learners' linguistic performance in terms of complexity, accuracy, and fluency may compete with each other (Sample and Michel, 2014; Sun and Révész, 2021). Particularly, it has been argued that a trade-off exists between attention to form and attention to meaning during task performance (Skehan, 1998, 2009; Skehan and Foster, 2001).

For communicative purposes, L2/FL learners are assumed to prioritize meaning (i.e., fluency) over form (i.e., accuracy and complexity) (Skehan, 2009). In other words, performing L2/FL tasks may lead to conflicts between meaning and form for learners' attentional resources. Therefore, when learners concentrate on being fluent in delivering the communicative content, fewer attentional resources will be available for producing complex and accurate utterances (Sample and Michel, 2014). Furthermore, following Skehan (2009), a further trade-off is likely to arise between these two latter dimensions because learners may lack resources to pay attention to both complexity and accuracy simultaneously.

As aforementioned, the incorporation of ASR-technology provides L2/FL learners with an avenue of repeated practice based on synchronic feedback, preparing themselves in advance for the in-class higher-order tasks. To the best of our knowledge, however, few studies have been conducted to investigate how the ASR technology influences EFL learners' linguistic performance, especially in the domain of vocabulary learning. It also remains unclear whether the trade-off effect still holds when ASR-based technology is incorporated into task-based language learning. The current study, therefore, aims to fill this gap by testing the Trade-off Hypothesis in the context of ASR-enhanced task-based language learning with a research focus on vocabulary learning. Based on the research gaps identified, three research questions (RQ) were formulated in the current study:

RQ 1: Does the ASR-based technology embedded in pre-class self-study lead to differences in EFL learners' lexical complexity in a flipped classroom?
RQ 2: Does the ASR-based technology embedded in pre-class self-study lead to differences in EFL learners' lexical accuracy in a flipped classroom?
RQ 3: Does the ASR-based technology embedded in pre-class self-study lead to differences in EFL learners' speed fluency in a flipped classroom?

## Methods

### Participants

Sixty-three first-year undergraduates of two EFL classes in a Chinese university were recruited in the quasi-experiment. Their majors included Chinese literature and arts, sociology, public administration and management, education, computer science and technology, biological engineering, law and mathematics. Their average age was 18.1 years old; 17.5% of them were male, and 82.5% were female. All the students consented to participate in the study approved by the research site university. According to the pre-intervention survey, the participants had English learning history for approximately 11 years on average and they reported an average score of 128.5 (out of 150) for their college entrance English examination, indicating that on a general basis they were ready to learn English at the tertiary level. With regard to their experiences of flipped learning, 90.5% of the students had “never” or “seldom” learned in a flipped fashion, and 9.5% “some” experience of flipped learning. Moreover, 65.1% of the participants reported “no” or “little” training specific for oral English, and 27% “some” experiences of oral English learning; only 7.9% had “sufficient” training in oral English back at high school.

### Course Design

This course was part of the College English program for Year 1 and Year 2 undergraduates, which aimed to develop learners' English proficiency and foster their English skills for both general and academic purposes. Each semester, the course covered a total of eight learning units. An online learning platform, i.e., Unipus (https://u.unipus.cn/), developed by the course book publisher, was utilized for the flipped implementation for both classes. All the course contents (i.e., vocabulary, cultural background information, texts and recordings, in-class tasks and post-class assignments) on Unipus were accessible with smart devices such as smartphones or tablets. On a weekly basis, the students in both classes had a 90-min face-to-face session with the same EFL teacher who had been teaching the program for ten consecutive years. Within each class, the students were randomly assigned into workgroups of three or four for performing group-based tasks, and for data collection reasons, the composition of the workgroups remained unchanged until the end of the semester.

**Figure 1:**
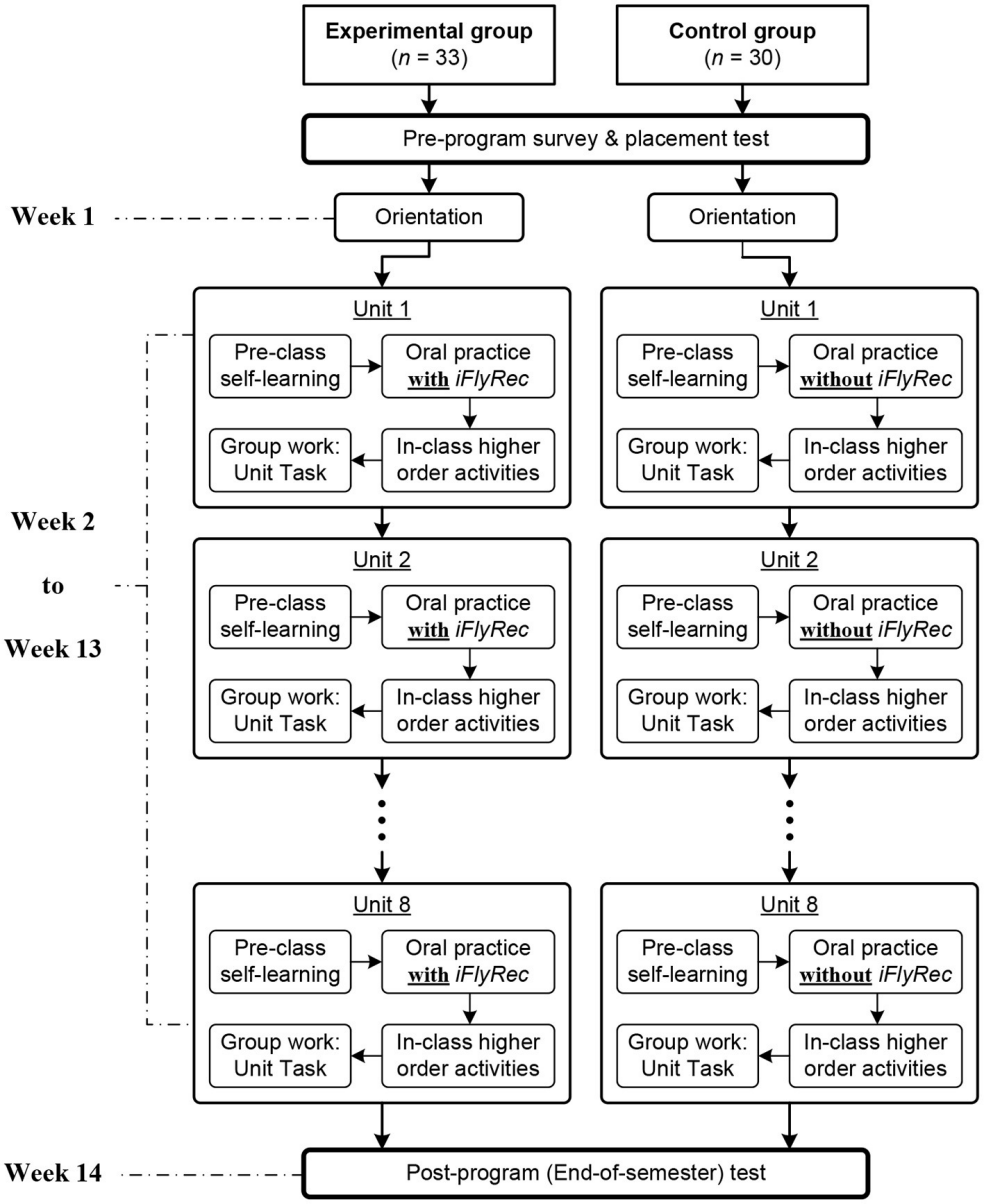
Instructional procedure.

Each learning unit consisted of several sections with varied learning tasks. According to Bloom's taxonomy (Anderson and Krathwohl, 2001), some of the sections and tasks were understanding- and remembering-oriented, such as *Reading Across Cultures* and *Language in Use*, while others were more applying-, analyzing-, evaluating-, and creating-oriented, such as *Reading Skills, Guided Writing* and *Unit Task (UT)*. In light of the rationale of flipped classroom approach, tasks that were at the lower level of the taxonomy (understanding- and remembering-oriented) were flipped outside the classroom for students' pre-class self-learning on Unipus. Conversely, tasks at the higher level (e.g., applying- and analyzing-oriented activities) were performed in class (Jong, 2019a; Jong et al., 2022). In particular, a comprehensive UT was performed in class toward the end of each learning unit. It was a production-oriented group activity for the students to conduct a topic-based discussion that required higher-order language skills such as analyzing and evaluating. The performance of each group member and their peer interaction while performing the UT were audio recorded as the major data source in this study. The instructional procedure is demonstrated in Figure 1.

**Figure 2:**
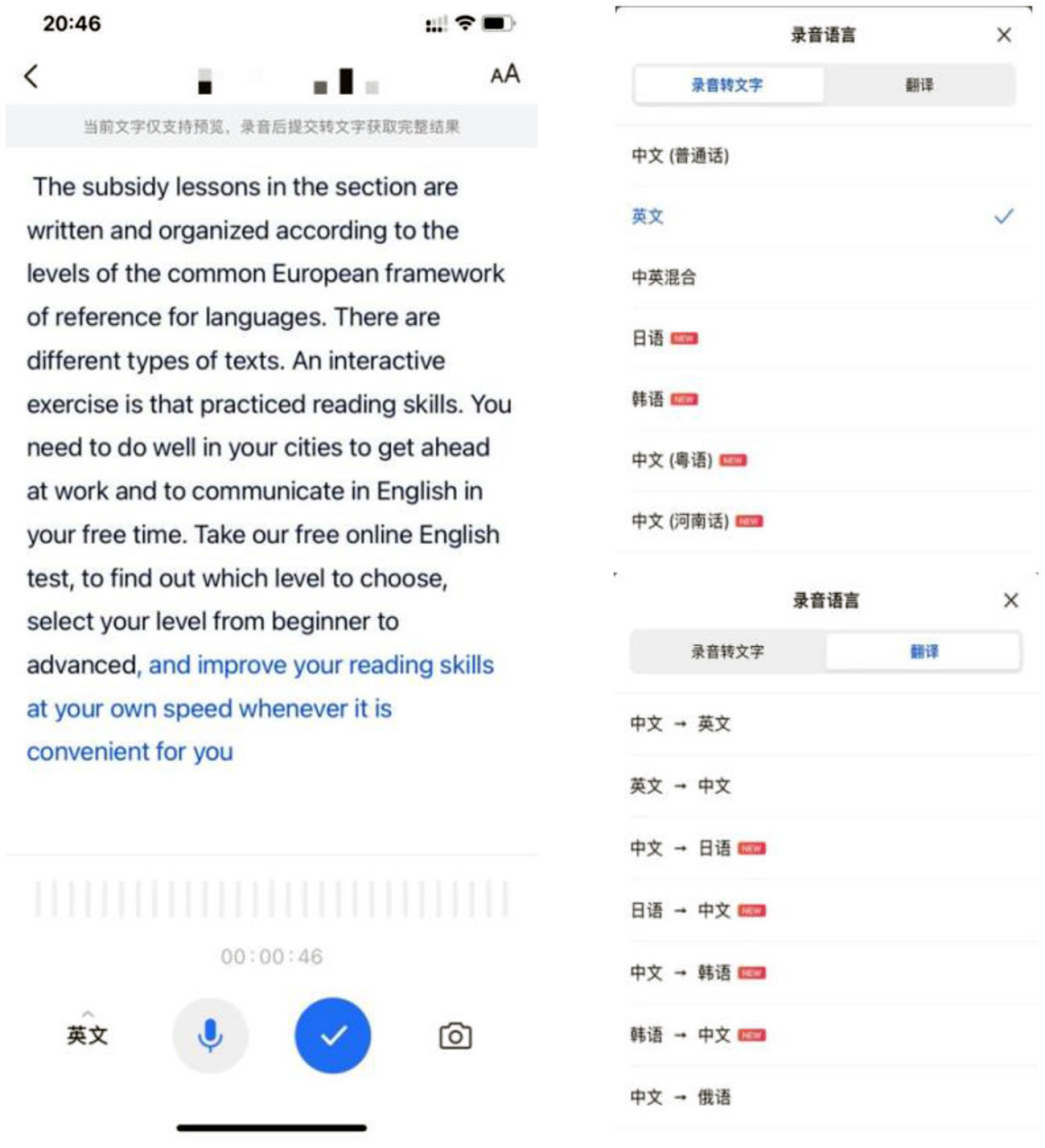
Screen capture of iFlyRec application. Reproduced with permission.

An ASR-based application called iFlyRec (https://www.iflyrec.com), which is developed by iFlyTek, a well-known intelligent speech and artificial intelligence company, was utilized in the study. iFlyRec is free to download and can run on iOS and Android systems. One of its salient features lies in its real-time conversion of speech into text in multiple languages and even some Chinese dialects (Figure 2). Moreover, it also supports interlingual translation in oral form in several languages including Chinese, English, Korean, Japanese, and Russian. In the current study, the students in the experimental group were required to perform oral tasks in pre-class self-learning with the assistance of iFlyRec. Based on the immediately transcribed texts as feedback for their oral speech, the experimental group students were encouraged to repeat their practice until their utterance was fully understood by the application. Contrarily, the students in the control group performed the same sets of pre-class oral tasks with no ASR-based applications. They needed to evaluate their oral performance by themselves. When they felt that their oral tasks were satisfactorily performed, the students in both groups should upload their recordings of their completed tasks to Unipus for assessment before the next class. Enlightened by the sociocultural theory, the integration of the ASR-based practices into students' pre-class self-learning is intended to promote their vocabulary learning, based on the ZPD assumption that there are gaps between learners' original level of language learning and their potential level of learning development when facilitated by social interaction with artifact/mediation. It is therefore hypothesized that with the instant feedback on language production afforded, the ASR technology could provide opportunities for mediated performance that can make a difference in vocabulary learning on the learners' side.

### Research Design

This study adopted a pre-and-post quasi-experimental design. The independent variable was the group factor of two levels, and the dependent variable was the participants' linguistic performance in relation to vocabulary learning (coded from their UT performance). The two classes were randomly determined as the experimental group (*n* = 33) and the control group (*n* = 30). A survey was administered before the experiment to gather the participants' background information and a placement test was used to measure their pre-intervention English proficiency, which was controlled for as a covariate in the data analysis. Accordingly, MANCOVA and mixed-design repeated measures ANCOVA procedures were adopted in this study to examine the between- and within-subjects effects.

Given that the participants might not know what was expected of them in a college EFL classroom when they just started college learning, their UT performance in Unit 1 was not collected in the study. Instead, their UT performance of Unit 2 was used as the pre-intervention data, and their performance of Unit 8 (i.e., the last unit of the semester) as the post-intervention data. The task performance of each workgroup was audio recorded while the students were performing the UTs. The recordings were transcribed into searchable text form and then coded with ELAN (https://tla.mpi.nl/tools/tla-tools/elan), a professional annotation tool for audio and video recordings. In data preprocessing, seven participants (four from the experimental group and three from the control group) were excluded because of their recording quality, dropout of the program or absence in class. Consequently, pre- and post-intervention recordings of 56 students (29 from the experimental group and 27 from the control group) were ultimately transcribed and coded for further analysis. The students were invited to proofread the transcriptions of their recordings to ensure the accuracy of the transcribed texts.

### Measures and Instruments

Based on the CAF framework, the participants' vocabulary learning performance was operationalized as lexical diversity, lexical accuracy and speed fluency (Table 1) in the current study. Specifically, lexical diversity was assessed through both simple metric (i.e., G-index) and complex metrics (i.e., vocd-D and MTLD), respectively. Lexical accuracy was quantified by the number of lexical errors against the analysis of speech unit (AS-unit), and speed fluency was estimated by unpruned speech rate, i.e., syllables per minute including all the utterances.

**Table 1:** Metrics for measuring vocabulary learning performance.

**CAF components**	**Sub-dimensions**	**Metrics**
Complexity	Lexical diversity	G-index, vocd-D, MTLD
Accuracy	Lexical accuracy	Lexical errors per AS-unit
Fluency	Speed fluency	Unpruned syllables articulated per minute

**Figure 3:**
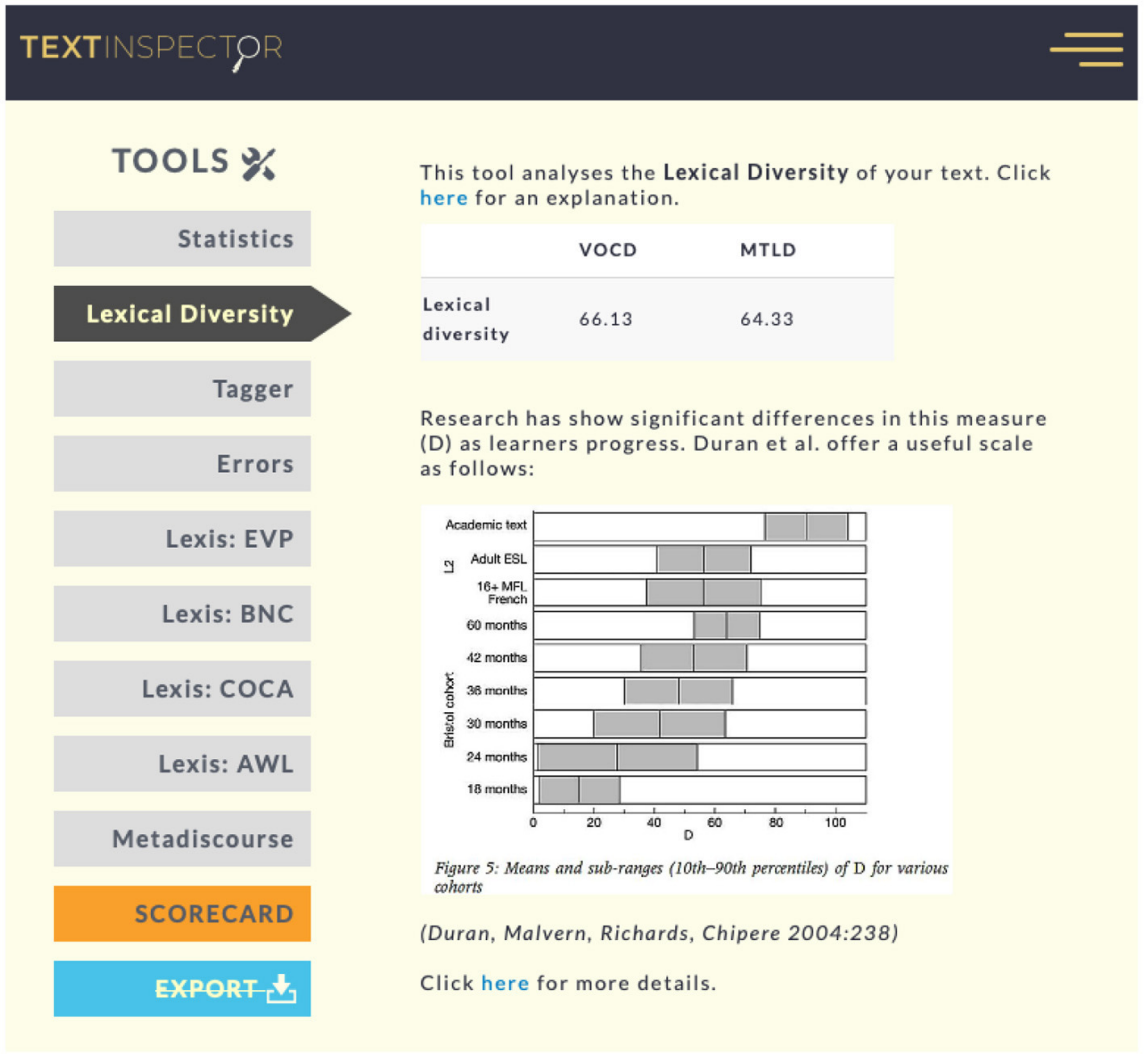
Screen capture of TextInspector. Reproduced with permission.

G-index, a widely utilized simple indicator of lexical complexity is obtained by dividing the types (the total number of different words) occurring in a speech or text sample by the square root of its tokens (the total number of words) (Guiraud, 1960). However, quantitative linguistic studies have shown that measures based on type/token ratio (TTR) are flawed and subject to the length of the text sample (see Richards and Malvern, 1997; Tweedie and Baayen, 1998, for a demonstration). In response, we employed two more complex metrics, i.e., vocd-D and the measure of textual lexical diversity (MTLD), which are based on mathematical probabilistic models and are not susceptible to text length. They are calculated through computer programs and are results of a series of random text samplings. The vocd-D value has been used in numerous studies, although deBoer (2014) cautioned that vocd-D was still affected by text length and might be less reliable outside of an ideal range of perhaps 100–500 words. Conversely, McCarthy and Jarvis (2007) demonstrated that MTLD was a powerful index of lexical diversity, but further research was needed to confirm their findings in a range of settings. As such, the present study adopted both metrics in the hope of obtaining a clearer picture of the data and avoiding drawing false conclusions. As was suggested by McCarthy and Jarvis (2010), researchers should use these indices together rather than any single index because lexical complexity can be assessed in many ways, and each approach may be informative as to the construct under investigation. Besides, the calculation of these two metrics involved all the words produced by the interlocutors (Albert, 2011). TextInspector (https://textinspector.com), a professional online tool for analyzing linguistic data, was utilized to calculate vocd-D and MTLD (Figure 3). It also provided basic statistics of a given text, such as TTR, syllable count, average sentence length and so on.

**Figure 4:**
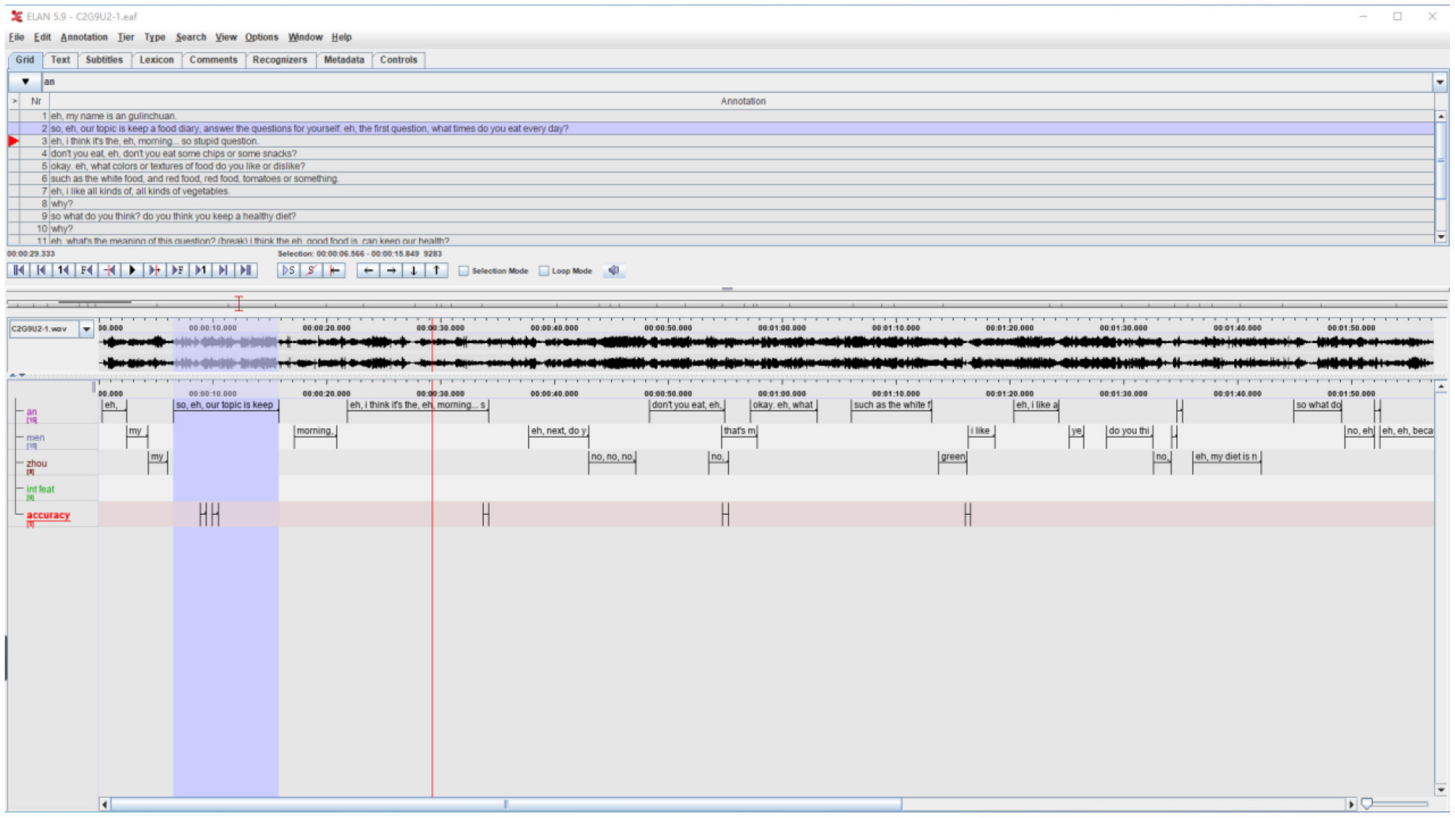
Screen capture of ELAN workspace. Reproduced with permission.

In terms of operationalizing lexical accuracy in this study, AS-unit was employed as the production unit, referring to “a single speaker's utterance consisting of an independent clause, or sub-clausal unit, together with any subordinate clause(s) associated with either” (Foster et al., 2000). It is a length-based production unit specifically proposed as an improved alternative for oral discourse segmentation in SLA (Norris and Ortega, 2009; Jiang et al., 2021). Compared with other production units in use (e.g., C-unit, T-unit; see Foster et al., 2000 for details), the AS-unit is adequate and reliable when applied to transcriptions of complex oral data, which tend not to lend themselves easily to a clear division into units (Foster et al., 2000), especially for non-native speakers of English. The lexical errors (e.g., retrieve inappropriate words or use them incorrectly in a specific context) were coded with ELAN, a piece of professional software for annotating audio and video recordings (Figure 4). One author and the course teacher conducted the coding and cross-checked the results. Any disagreement between the two coders was resolved through discussion until a consensus was reached. The current study was part of a doctoral study that involved more CAF metrics, and the overall inter-rater reliability was estimated through Krippendorff's α (Hayes and Krippendorff, 2007) and was computed to be 0.818 (>0.8), indicating consistency between the two coders.

The speed fluency was estimated by unpruned speech rate (i.e., syllables per minute including all the utterances such as false starts, self-corrections, and repetitions), which was computed by dividing the number of all the syllables produced by the time taken to produce them. As aforementioned, the count of syllables was reported as a basic statistic by TextInspector, and the time taken for each interlocutor could be easily read through ELAN after annotating the audio clips.

## Results

### Lexical Complexity

Lexical complexity was estimated through G-index, vocd-D value and MTLD, of which the descriptive results were tabulated below (Table 2). MANCOVA was performed to examine the between-subjects effects with the pre-intervention placement test score as a covariate. Results showed that at the significance level of 0.05 (‘^*^’ indicates *p* < 0.05, ‘^**^’ *p* < 0.01, ‘^***^’ *p* < 0.001), the two groups had no significant differences in any of the three metrics of lexical diversity when performing their first UT. Conversely, after the intervention of a semester, the students in the experimental group significantly outscored their counterparts in the control group on G-index (*F* = 6.571^*^; *p* = 0.013 < 0.05); vocd-D (*F* = 12.502^***^; *p* < 0.001), and MTLD (*F* = 4.627^*^; *p* = 0.036 < 0.05) when performing the last UT. The corresponding effect sizes (estimated by partial η^2^) were calculated to be 0.110 for G-index, 0.191 for vocd-D and 0.080 for MTLD, respectively, which all indicated medium to large effect sizes of the intervention on students' lexical diversity. Following Cohen (1988) and Miles and Shevlin (2001), the thresholds of partial η^2^ adopted in this study are small partial η^2^ > 0.01, medium > 0.06, and large > 0.14.

**Table 2:** Descriptive statistics.

**Metric**	**Group**	**Pre-intervention mean**	**Post-intervention mean**	***n***
G-index	EG	5.60	6.54	29
	CG	5.52	6.10	27
vocd-D	EG	45.36	51.85	29
	CG	38.27	40.14	27
MTLD	EG	32.35	38.07	29
	CG	25.98	28.35	27

*EG, experimental group; CG, control group*.

**Figure 5:**
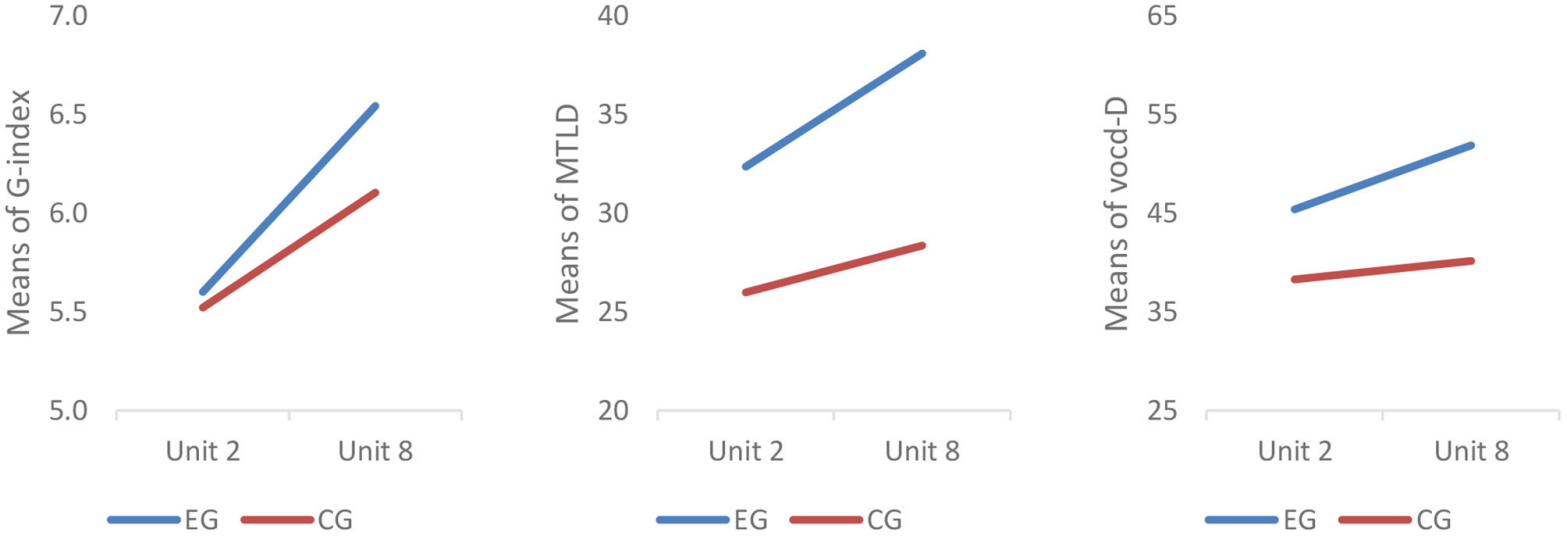
Profile plot of lexical diversity metrics. Covariates appearing in the model are evaluated at the following values: placement test score = 83.05.

Mixed-design repeated measures ANCOVAs were performed to further examine the corresponding between- and within-subjects effects. The results showed a significant between-subjects effect on vocd-D (*F* = 5.744^*^; *p* = 0.020 < 0.05) and MTLD (*F* = 4.293^*^; *p* = 0.043 < 0.05). Conversely, no significant between-subjects effect was noticed on G-index (*F* = 2.691^*^; *p* = 0.107 > 0.05). Follow-up simple-effect tests revealed that the experimental group had a significant improvement on G-index (*t* = 7.994^***^, *p* < 0.001) and MTLD (*t* = 2.271^*^, *p* = 0.031 < 0.05) and a marginally significant improvement on vocd-D (*t* = 1.914, *p* = 0.066 < 0.1). Conversely, in the control group, only the improvement on G-index was statistically significant (*t* = 5.051 ^***^p < 0.001); no statistically significant change was witnessed on vocd-D (*t* = 0.944, *p* = 0.354 >0.05) or MTLD (*t* = 1.345, *p* = 0.190 > 0.05) (Figure 5).

### Lexical Accuracy

Lexical accuracy was quantified through an error-based metric, i.e., the number of lexical errors per AS-unit. Descriptive statistics showed that before the intervention, the students in the experimental group generated 0.204 lexical errors per AS-unit and their counterparts in the control group 0.146 lexical errors per AS-unit. After the intervention, the number of lexical errors per AS-unit of the experimental group decreased to 0.156 and that of the control group dropped to 0.140. However, the results of MANCOVA revealed that there was neither significant difference of lexical accuracy between their pre-intervention performance (*F* = 1.022; *p* = 0.317 > 0.05) nor their post-intervention performance (*F* = 0.001; *p* = 0.980 > 0.05).

**Figure 6:**
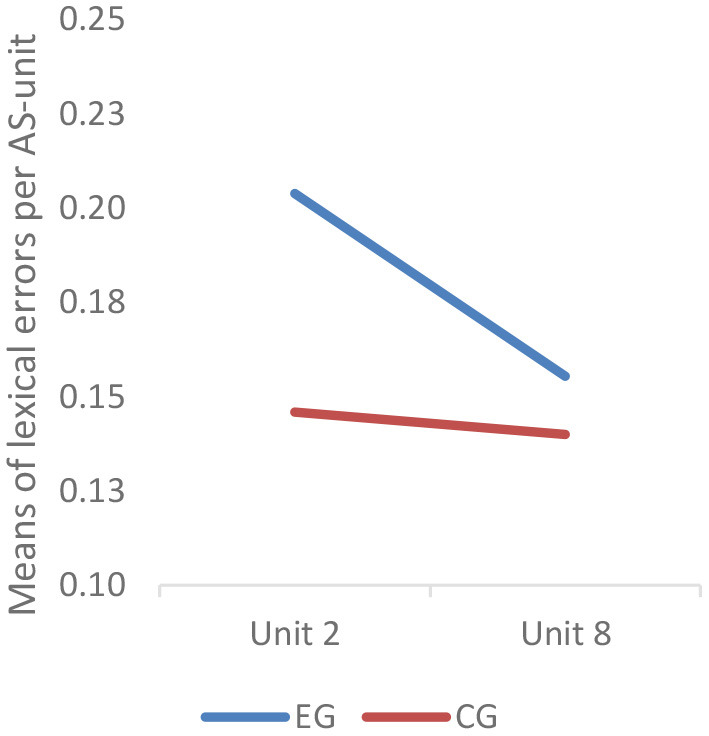
Profile plotsof lexical accuracy. Covariates appearing in the model are evaluated at the following values: placement test score = 83.05.

The results of mixed-design repeated measures ANCOVA indicated that the between-subjects effect was not statistically significant (*F* = 0.339; *p* = 0.563 > 0.05), although graphically the experimental group appeared to have a more salient drop in lexical errors per AS-unit (Figure 6). Simple-effect tests also revealed no significant change over time in either the experimental group (*t* = 1.333; *p* = 0.193 > 0.05) or the control group (*t* = 0.117; *p* = 0.908 > 0.05).

### Speed Fluency

Speed fluency was estimated by unpruned speech rate (i.e., syllables per minute including all the utterances such as false starts, self-corrections, and repetitions), which was computed by dividing the number of all the syllables produced by the time taken to produce them. Descriptive statistics showed that before intervention, the unpruned speech rate of the students in the experimental group was 147.44 syllables per minute, while that of the students in the control group was 137.25. The MANCOVA results showed that there was no significant difference between the two groups (*F* = 2.555; *p* = 0.116 > 0.05). However, after the intervention, the unpruned speech rate of the students in the experimental group increased to 157.81 and that of the control group students increased to 139.56, indicating a seemingly limited improvement for the control group. The results of MANCOVA revealed that the experimental group outperformed their control group counterparts significantly (*F* = 6.322^*^; *p* = 0.015 < 0.05) with a medium to large effect size (partial η^2^ = 0.107) (Cohen, 1988; Miles and Shevlin, 2001).

**Figure 7:**
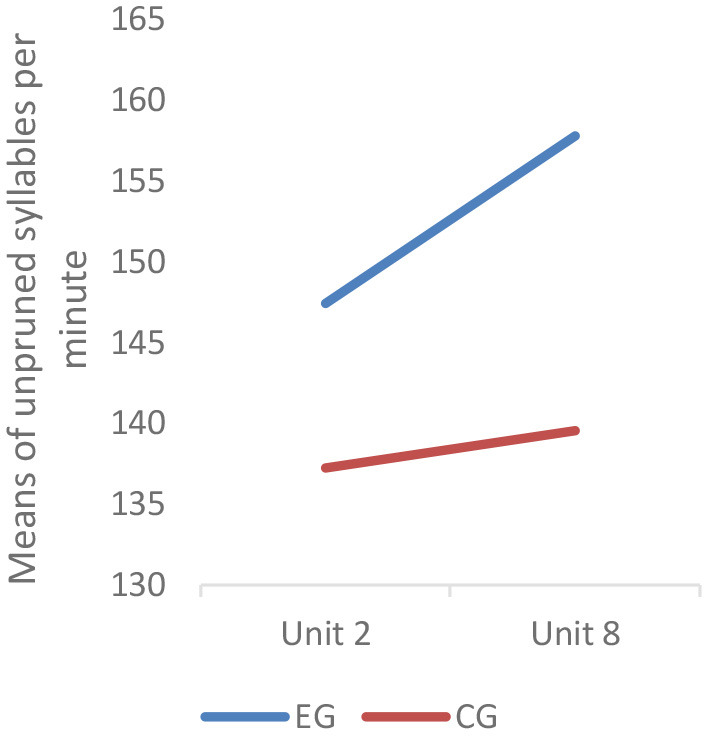
Profile plot of speed fluency. Covariates appearing in the model are evaluated at the following values: placement test score = 83.05.

Likewise, the between- and within-subjects effects were also examined through mixed design repeated measures ANCOVA. A significant between-subjects effect was witnessed (*F* = 5.786^*^; *p* = 0.02 < 0.05). With regard to the within-subjects effect, respective simple-effect tests were performed, and the results showed that a significant improvement in speed fluency was seen in the experimental group (*t* = 2.180^*^; *p* = 0.038 < 0.05), while the control group did not demonstrate a statistically significant improvement in their speed fluency (*t* = −0.392; *p* = 0.698 > 0.05) (Figure 7).

## Discussion

The results showed that the experimental group students outscored their control group counterparts on lexical complexity (i.e., G-index, vocd-D and MTLD) and speed fluency. But on lexical accuracy, there was no significant post-intervention difference between the two groups. In terms of the within-subjects effect, the experimental group had significant improvement on all three metrics of lexical complexity and speed fluency. In contrast, the control group only had significant improvement on G-index. On lexical accuracy, no significant within-subjects effect was observed in either group.

Generally, the results of this study confirmed the positive effects of integrating the ASR-based application on EFL students' learning, corroborating previous findings (e.g., Evers and Chen, 2020; Dai and Wu, 2021; Jiang et al., 2021). The findings also supported earlier empirical claims that iCALL technologies could provide opportunities for effective vocabulary learning (Chen and Hsu, 2019; Li and Hafner, 2021). It is well-acknowledged that learner preparedness in a flipped classroom plays a pivotal role in students' engagement and task performance in class (Sun and Xie, 2020). Theoretically, owing to the course teachers' pedagogical design, the pre-class self-study in a flipped setting is supposed to be well-organized (Lee and Choi, 2019). However, in practice, students' self-learning is usually affected by factors such as inadequate self-regulated learning ability, resulting in less productive and inefficient preparation for class (Jiang et al., 2020). In the present study, although both groups were learning in a flipped fashion, the significant between-subjects differences indicated that the integration of the ASR-based practice was more goal-oriented and conducive to preparing the students for higher-order interactive tasks in class. Particularly, the ASR-based oral practice featured high interactivity in tandem with synchronic feedback, thus providing the EFL students with ample opportunities to correct themselves. Apart from serving as immediate feedback on the students' utterances, the transcribed texts also visualized students' gradual improvement each time they practiced with self-correction, giving the students an instant sense of accomplishment. It corresponded with the *gradual* feature of effective feedback proposed by Aljaafreh and Lantolf (1994) that would best promote learners' ZPD from a sociocultural perspective.

Specifically, findings of RQ 1 revealed that benefited from the use of the ASR-based application, the experimental group outscored their counterparts in the control group on G-index, vocd-D, and MTLD in the post-intervention performance, and there was significant within-subjects growth observed on all the three metrics in the experimental group. Measures such as vocd-D and MTLD are deemed critical indicators of L2/FL lexical proficiency, as learners with a richer and more diverse vocabulary are considered as more lexically proficient (Crossley et al., 2009). The experimental group's significant improvement in lexical complexity may be attributed to the ample opportunities for practice made available with the visualized feedback provided by the ASR-based application (Jiang et al., 2021). Before each class, the students in the experimental group performed ASR-enhanced oral tasks which allowed them to correct themselves using the transcribed texts repeatedly. With the aid of the social artifact, they might become more aware of their word choices as they could clearly see every word they uttered while practicing, leading to their deliberate avoidance of a repetitive word used in their previous utterances. In other words, they might variate their use of vocabulary when practicing with the ASR-based application to express themselves. This may well reflect the developmental process from other-regulation to self-regulation as indicated by the sociocultural theory (Wertsch, 1979). In other words, the scaffold afforded by the ASR-based application facilitated the regulation of learners' vocabulary use, which could gradually lead to the internalization of the regulation so that learners became able to self-regulate their vocabulary choice even when such scaffold was released. In fact, this echoes the premise of self-regulated education that the provision of adequate learning scaffolds is always salient in the course of self-regulated learning (Jong, 2019b; Dong et al., 2020). Moreover, the ASR-based practice created an avenue for students to employ the newly learned words in pre-class self-study and hence consolidated their retention for later use. In group-based discussion, their group members also used some of these words, which further reinforced their grip on these lexical gains. As was argued by El Majidi et al. (2021), such a cyclic lexical process might enable the students to incrementally build a diverse and rich lexicon. Additionally, improvement in vocabulary also seemed to allow more working memory to retrieve more sophisticated vocabulary, therefore producing more lexically diverse and complex speech.

The findings in response to RQ 2 revealed that the two groups had no significant difference in lexical accuracy and neither group had significant gains of lexical accuracy over time, although descriptive statistics showed that the students in the experimental group made fewer lexical errors in the post-intervention UT. This may be attributable to the dictation nature of the ASR technology used in this study. iFlyRec is a speech-to-text dictation ASR application developed for native speakers. Although it was argued that dictation ASR could be more effective in enhancing students' foreign language oracy when combined with scaffolded activities (Evers and Chen, 2020), since it was not designed for pedagogical purposes, dictation ASR-based applications do not provide as sufficient intended feedback on learners' speech as interactive ASR applications such as Google Assistant (Tai and Chen, 2020). This is one of the demerits of dictation ASR technology. Additionally, the role of body language or human emotion in their speech may not be considered in dictation ASR-based oral practice, although they are indispensable elements in human communication. To bridge the limitations of dictation ASR, future studies may combine the use of iFlyRec together with Google Assistant to see whether the two kinds of ASR technologies could jointly improve EFL learners' oracy and further enhance iCALL-based EFL pedagogy.

Contrastingly, the findings of RQ 3 uncovered that significant between- and within-subjects effects in the experimental students' speed fluency were also witnessed in this study. Since the students in the experimental group were encouraged to repeatedly perform the ASR-based tasks (i.e., a condition of task repetition), their pre-class self-study might result in a solid practice effect, which could further lead to a higher degree of automaticity in their utterances when performing the UT. Following Tavakoli et al. (2016), such automaticity is manifested in flow, continuity and smoothness of speech. The automaticity in learners' oral production also coincided with Vygotsky's concept of internalization, which could be interpreted as transforming cognitive functions that are once performed through sociocultural mediation by artifacts and self into cognitive abilities that can be performed independently (Lantolf and Thorne, 2006). Some empirical studies have evidenced that task repetition served as a factor contributing to oral L2/FL fluency (e.g., Ahmadian and Tavakoli, 2011; Goh, 2017). Specifically, the task repetition in this study is regarded as a condition of content repetition, which according to previous studies, tends to be more advantageous for linguistic fluency at the cost of grammatical accuracy (Patanasorn, 2010). On the other hand, the immediate feedback in its written form provided by the ASR-based application might have enhanced the experimental group students' preparedness for developing a greater degree of automatization in their performance (DeKeyser, 2001, 2007; Segalowitz, 2010; Jiang et al., 2021). Therefore, the pedagogical intervention of ASR-based oral tasks that enabled sustained practice could assist the students in successfully managing their discourse flow when performing the higher-order in-class tasks.

Furthermore, the findings of this study lent some support to Skehan's Trade-off Hypothesis. The triarchic CAF framework generally defines language proficiency as the complex interplay of the three constructs, i.e., complexity, accuracy, and fluency (Tavakoli, 2016), which may be distinctively manifested under different conditions of L2/FL use. The three constructs may be differentially developed by different types of learners and under different learning conditions (Housen et al., 2012). In the current study, the incorporation of ASR-based application for oral practice led to significant growth in the students' speed fluency, while no significant improvement was observed in their lexical accuracy. The contrasting results may indicate a conflict of attention to form and attention to meaning on the learner's side. To be specific, the participants in this study might focus more on the expression of ideas when performing the group-based discussion, indicating a possible priority of meaning over form in oral speech. The practice effect produced by the ASR-based practice seemingly resulted in a degree of proceduralization based on oral lexical chunks, which further led L2/FL learners to develop a state of automatization (DeKeyser, 2001, 2007; Segalowitz, 2010). “When appropriate lexical chunks are readily available, fewer searches are needed, therefore accelerating the formulation process resulting in greater fluidity in oral production” (El Majidi et al., 2021, p. 13). Therefore, in essence, ASR-based technology is regarded as a tool for enhancing L2/FL learners' meaning-oriented proficiency more than form-oriented, indicating that learners may increase their oral fluency at the cost of accuracy.

On the other hand, as was claimed by Skehan (2009), a sequent trade-off might occur between the form-related constructs, i.e., complexity and accuracy, probably because the students were incapable of paying attention to both constructs simultaneously. This result was in line with the previous studies witnessing trade-offs between complexity and accuracy (e.g., Sample and Michel, 2014; Rashtchi and Yousefi, 2017; Granena and Yilmaz, 2019). In the current study, the UTs were considered higher-order tasks that required comprehensive English proficiency, and following Kim (2015), those complex tasks may demand more attentional resources to content, thus allowing less attention allotted to language forms. Although the tripartite CAF conceptualization of L2 performance has become standardized and widely accepted in task-based language teaching (TBLT) (Bui, 2021), the complicated interplay among them remains underexplored in the field of iCALL. Accordingly, more empirical investigations are desirable on this issue in order to understand the interrelationships between the three constructs and how technologies may affect their interplay.

## Conclusions and Implications

The current study investigated the effects of the ASR-based technology on EFL students' vocabulary learning based on a pre- and post-intervention quasi-experiment. It was found that the integration of ASR-based technology resulted in significant between-subjects effects on lexical complexity (i.e., G-index, vocd-D, and MTLD) and speed fluency. Conversely, the between-subjects effect on lexical accuracy was not significant. In terms of the within-subjects effect, the experimental group had significant growth on all the three metrics of lexical complexity and speed fluency, while the control group only had significant improvement on G-index. No significant within-subjects effect was seen in either group on lexical accuracy. Given the improvement in EFL students' speed fluency and lexical complexity while not in their lexical accuracy, Skehan's Trade-off Hypothesis was supported in this study.

Pedagogically, the integration of the ASR technology into a flipped foreign language classroom alters the general notion of pre-class self-study in a flipped foreign language classroom which is primarily passive absorption of factual knowledge through pre-recorded video clips on the learner's side. With the ASR-based application, the pre-class oral practice allows the flipped EFL pedagogy to include an active component which provides immediate feedback for students' self-study, thus making it no longer a passive reception of knowledge. Therefore, the ASR-enhanced oral practice can shed light on the pedagogical design of a flipped foreign language classroom. When in-class time is repurposed for higher order language skills in a flipped classroom, the ASR-based technology can be a useful tool for speaking practice, especially when students have limited opportunities to receive feedback on their speaking performance from proficient or native speakers (McCrocklin, 2019).

## Limitations

Despite the measurable effects of the ASR-based technology on the participants' linguistic performance, the results of this study should be treated with caution due to the following limitations. First, the participants in this study were only enrolled in one university in Chinese mainland, which might raise concerns with the representativeness of the sample. Therefore, more empirical studies conducted in similar research contexts are needed to examine the effects of ASR technology on EFL learners' vocabulary learning. Meanwhile, given that there are studies reporting no evidence for Skehan's Trade-off Hypothesis, EFL teachers need to be cautious about the trade-off in pedagogical practice (Lan et al., 2018). Second, due to the complicated conceptualization and measuring system of the CAF constructs, CAF studies always raise concerns with the operationalizations of multi-dimensional CAF constructs. For example, lexical density and lexical sophistication might be added to the study as another means of measuring lexical complexity. While so far, no synthesis work has been conducted to scope the studies pertaining to the use of ASR technology in L2/FL learning, follow-up studies may need to employ more comprehensive metrics to perceive CAF as a dynamic and interrelated set of constantly changing subsystems (Norris and Ortega, 2009) in the domain of iCALL. Third, since the Chinese EFL learning context is crucial in understanding the students' EFL learning behavior and in-class peer interaction, a mixed method approach could be employed in future research to draw a holistic picture of how the factors with respect to the Chinese context such as the Chinese educational practice and the local Chinese culture may influence students' in-class task-based oral performance. Fourth, due to the outbreak of the COVID-19 Pandemic, a delayed post-test was not conducted to explore the delayed effects of the ASR technology on the students' vocabulary learning performance. Future studies are advised to perform delayed test to see whether the use of the dictation ASR application has a long-term effect on EFL learners' vocabulary learning.

## Data Availability Statement

The raw data supporting the conclusions of this article will be made available by the authors, without undue reservation.

## Ethics Statement

The studies involving human participants were reviewed and approved by Survey and Behavioral Research Ethics Committee, the Chinese University of Hong Kong. The patients/participants provided their written informed consent to participate in this study.

## Author Contributions

Material preparation, data collection, and analysis were performed by MY-CJ, MS-YJ, C-SC, WW-FL, NW, BS, and BH. The first draft of the manuscript was written by MY-CJ and BS. All authors contributed to the study conception and design, commented on previous versions of the manuscript, read, and approved the final manuscript.

## Funding

This work was funded by Fuzhou University Scientific Research Project Fund (Award No. XRC202203).

## Conflict of Interest

The authors declare that the research was conducted in the absence of any commercial or financial relationships that could be construed as a potential conflict of interest.

## Publisher's Note

All claims expressed in this article are solely those of the authors and do not necessarily represent those of their affiliated organizations, or those of the publisher, the editors and the reviewers. Any product that may be evaluated in this article, or claim that may be made by its manufacturer, is not guaranteed or endorsed by the publisher.

## References

[B1] Ågren M. Granfeldt J. Schlyter S (2012). “The growth of complexity and accuracy in L2 French,” in Dimensions of L2 Performance and Proficiency: Investigating Complexity, Accuracy and Fluency in SLA, eds A. Housen, F, Kuiken and I. Vedder (Amsterdam: John Benjamins), 95–119. 10.1075/lllt.32.05agr

[B2] Abdulrahman T. R. Jullian M. H. (2020). Engaging young learners in learning vocabulary: a study on learners' perception. Akademika 9, 139–153. 10.34005/akademika.v9i01.805

[B3] Ahmadian M. J. Tavakoli M. (2011). The effects of simultaneous use of careful online planning and task repetition on accuracy, complexity, and fluency in EFL learners' oral production. Lang. Teach. Res. 15, 35–59. 10.1177/1362168810383329

[B4] Albert A. (2011). “When individual differences come into play: the effect of learner creativity on simple and complex task performance,” in Second Language Task Complexity, ed P. Robinson (Amsterdam: John Benjamins), 239–265. 10.1075/tblt.2.16ch9

[B5] Aljaafreh A. Lantolf J. P. (1994). Negative feedback as regulation: second language learning in the zone of proximal development. Mod. Lang. J. 78, 465–483. 10.1111/j.1540-4781.1994.tb02064.x

[B6] Anderson L. W. Krathwohl D. R. (2001). A Taxonomy for Learning, Teaching, and Assessing: A Revision of Bloom's Taxonomy of Educational Objectives. New York, NY: Addison Wesley Longman.

[B7] Barrot J. Gabinete M. K. (2021). Complexity, accuracy, and fluency in the argumentative writing of ESL and EFL learners. Int. Rev. Appl. Linguist. Lang. Test. 59, 209–232. 10.1515/iral-2017-0012

[B8] Bashori M. van Hout R. Strik H. Cucchiarini C. (2020). Web-based language learning and speaking anxiety. Comput. Assist. Lang. Learn. 1−32. 10.1080/09588221.2020.1770293

[B9] Bashori M. van Hout R. Strik H. Cucchiarini C. (2021). Effects of ASR-based websites on EFL learners' vocabulary, speaking anxiety, and language enjoyment. System 99,102496. 10.1016/j.system.2021.102496

[B10] Bui G. (2021). Influence of learners' prior knowledge, L2 proficiency and pre-task planning on L2 lexical complexity. Int. Rev. Appl. Linguist. Lang. Test. 59, 543–567. 10.1515/iral-2018-0244

[B11] Bulté B. Housen A. (2012). “Defining and operationalising L2 complexity,” in Dimensions of L2 Performance and Proficiency: Investigating Complexity, Accuracy and Fluency in SLA, eds A. Housen, F. Kuiken and I. Vedder (Amsterdam: John Benjamins), 21–46. 10.1075/lllt.32.02bul

[B12] Bulté B. Roothooft H. (2020). Investigating the interrelationship between rated L2 proficiency and linguistic complexity in L2 speech. System 91,102246. 10.1016/j.system.2020.102246

[B13] Chambers F. (1997). What do we mean by oral fluency? System. 25, 535–544. 10.1016/S0346-251X(97)00046-8

[B14] Chavez M. (2014). Variable beliefs about the need for accuracy in the oral production of German: an exploratory study. Int. J. Appl. Linguist. 24, 97–127. 10.1111/ijal.12029

[B15] Chen H. J. H. Hsu H. L. (2019). The impact of a serious game on vocabulary and content learning. Comput. Assist. Lang. Learn. 33, 811–832. 10.1080/09588221.2019.159319735319480

[B16] Cohen J. (1988). Statistical Power Analysis for the Behavioral Sciences, 2nd Edn. Erlbaum.

[B17] Crossley S. Salsbury T. McNamara D. (2009). Measuring L2 lexical growth using hypernymic relationships. Lang. Learn. 59, 307–334. 10.1111/j.1467-9922.2009.00508.x

[B18] Dai Y. J. Wu Z. W. (2021). Mobile-assisted pronunciation learning with feedback from peers and/or automatic speech recognition: a mixed-methods study. Comput. Assist. Lang. Learn.1–24. 10.1080/09588221.2021.1952272

[B19] deBoer F. (2014). Evaluating the comparability of two measures of lexical diversity. System 47, 139–145. 10.1016/j.system.2014.10.008

[B20] DeKeyser R. (2001). “Automaticity and automatization,” in Cognition and Second Language Instruction, ed P. Robinson (New York, NY: Cambridge University Press), 125–151. 10.1017/CBO9781139524780.007

[B21] DeKeyser R. (2007). “Situating the concept of practice,” in Practicing in a Second Language: Perspectives From Applied Linguistics and Cognitive Psychology, ed R. DeKeyser (New York, NY: Cambridge University Press), 1–18. 10.1017/CBO9780511667275.002

[B22] Dong A. M. Jong M. S. Y. King R. (2020). How does prior knowledge influence learning engagement? The mediating roles of cognitive load and help-seeking. Front. Psychol. 11,591203. 10.3389/fpsyg.2020.591203 PMC7658369 33192933

[B23] El Majidi A. de Graaff R. Janssen D. (2021). Debate as a pedagogical tool for developing speaking skills in second language education. Lang. Teach. Res. 1–22. 10.1177/13621688211050619

[B24] Ellis R. (2003). Task-Based Language Learning and Teaching. Oxford: Oxford University Press.

[B25] Ellis R. (2008). The Study of Second Language Acquisition, 2nd Edn. Oxford: Oxford University Press.

[B26] Ellis R. (2009). Corrective feedback and teacher development. L2 J. 1, 3–18. 10.5070/L2.V1I1.9054

[B27] Evers K. Chen S. (2020). Effects of an automatic speech recognition system with peer feedback on pronunciation instruction for adults. Comput. Assist. Lang. Learn. 1–21. 10.1080/09588221.2020.1839504

[B28] Foster P. Tonkyn A. Wigglesworth G. (2000). Measuring spoken language: a unit for all reasons. Appl. Linguist. 21, 354–375. 10.1093/applin/21.3.354

[B29] Franco H. Bratt H. Rossier R. Rao Gadde V. Shriberg E. Abrash V.. (2010). EduSpeak®: a speech recognition and pronunciation scoring toolkit for computer-aided language learning applications. Lang. Test. 27, 401–418. 10.1177/0265532210364408

[B30] Goh C. C. (2017). Research into practice: scaffolding learning processes to improve speaking performance. Lang. Teach. 50, 247–260. 10.1017/S0261444816000483

[B31] Granena G. Yilmaz Y. (2019). Phonological short-term memory capacity and L2 oral performance. J. Second Lang. Stud. 2, 317–335. 10.1075/jsls.19005.gra28025804

[B32] Guiraud P. (1960). Problèmes et Méthodes de la Statistique Linguistique. Paris: Presses universitaires de France.

[B33] Han Y. X. Zhao S. Ng L. L. (2021). How technology tools impact writing performance, lexical complexity, and perceived self-regulated learning strategies in EFL academic writing: a comparative study. Front. Psychol. 12:752793. 10.3389/fpsyg.2021.752793 34803833 PMC8596490

[B34] Hayes A. F. Krippendorff K. (2007). Answering the call for a standard reliability measure for coding data. Commun. Methods Meas. 1, 77–89. 10.1080/19312450709336664

[B35] Housen A. Kuiken F. (2009). Complexity, accuracy, and fluency in second language acquisition. Appl. Linguist. 30, 461–473. 10.1093/applin/amp048

[B36] Housen A. Kuiken F. Vedder I. (2012). “Complexity, accuracy and fluency: definitions, measurement and research,” in Dimensions of L2 Performance and Proficiency: Investigating Complexity, Accuracy and Fluency in SLA, eds A. Housen, F, Kuiken and I. Vedder (Amsterdam: John Benjamins), 1–20. 10.1075/lllt.32.01hou

[B37] Jiang M. Y. C. Jong M. S. Y. Lau W. W. F. Chai C. S. (2021). Using automatic speech recognition technology to enhance EFL learners' oral language complexity in a flipped classroom. Aust. J. Educ. Technol. 37, 110–131. 10.14742/ajet.6798

[B38] Jiang M. Y. C. Jong M. S. Y. Lau W. W. F. Chai C. S. Liu K. S. X. Park M. (2020). A scoping review on flipped classroom approach in language education: challenges, implications and an interaction model. Comput. Assist. Lang. Learn. 1–32. 10.1080/09588221.2020.1789171

[B39] Jong M. S. Y. (2017). Empowering students in the process of social inquiry learning through flipping the classroom. Educ. Technol. Soc. 20, 306–322.

[B40] Jong M. S. Y. (2019a). To flip or not to flip: social science faculty members' concerns about flipping the classroom. J. Comput. High. Educ. 31, 391–407. 10.1007/s12528-019-09217-y

[B41] Jong M. S. Y. (2019b). Sustaining the adoption of gamified outdoor social enquiry learning in high schools through addressing teachers' emerging concerns: a three-year study. Br. J. Educ. Technol. 50, 1275–1293. 10.1111/bjet.12767

[B42] Jong M. S. Y. Chen G. Tam V. Hue M. T. Chen M. (2022). Design-based research on teacher facilitation in a pedagogic integration of flipped learning and social enquiry learning. Sustainability 14:996. 10.3390/su14020996

[B43] Jong M. S. Y. Chen G. W. Tam V. Chai C. S. (2019). Adoption of flipped learning in social humanities education: the FIBER experience in secondary schools. Interactive Learn. Environ. 27, 1222–1238. 10.1080/10494820.2018.1561473

[B44] Kim I. S. (2006). Automatic speech recognition: Reliability and pedagogical implications for teaching pronunciation. Educ. Technol. Soc. 9, 322–334.

[B45] Kim Y. (2015). “The role of tasks as vehicles for language learning in classroom interaction,” in The Handbook of Classroom Discourse and Interaction, ed N. Markee (West Sussex: John Wiley and Sons), 163–181. 10.1002/9781118531242.ch10

[B46] Kormos J. Dénes M. (2004). Exploring measures and perceptions of fluency in the speech of second language learners. System 32, 145–164. 10.1016/j.system.2004.01.001

[B47] Lambert C. Kormos J. (2014). Complexity, accuracy, and fluency in task-based L2 research: toward more developmentally based measures of second language acquisition. Appl. Linguist. 35, 607–614. 10.1093/applin/amu047

[B48] Lan Y. J. Botha A. Shang J. J. Jong M. S. Y. (2018). Technology-enhanced contextual game-based language learning. Educ. Technol. Soc. 21, 86–89.

[B49] Lantolf J. P. Thorne S. L. (2006). Sociocultural Theory and the Genesis of Second Language Development. Oxford: Oxford University Press.

[B50] Lee J. Choi H. (2019). Rethinking the flipped learning pre-class: its influence on the success of flipped learning and related factors. Br. J. Educ. Technol. 50, 934–945. 10.1111/bjet.12618

[B51] Lennon P. (1990). Investigating fluency in EFL: a quantitative approach. Lang. Learn. 40, 387–417. 10.1111/j.1467-1770.1990.tb00669.x

[B52] Levis J. (2007). Computer technology in teaching and researching pronunciation. Annu. Rev. Appl. Linguist. 27, 184–202. 10.1017/S0267190508070098

[B53] Li Y. Hafner C. A. (2021). Mobile-assisted vocabulary learning: investigating receptive and productive vocabulary knowledge of Chinese EFL learners. ReCALL. 34, 66–80. 10.1017/S0958344021000161

[B54] Liao J. L. (2020). Do L2 lexical and syntactic accuracy develop in parallel? Accuracy development in L2 Chinese writing. System 94:102325. 10.1016/j.system.2020.102325

[B55] Lo Y. Y. Murphy V. A. (2010). Vocabulary knowledge and growth in immersion and regular language-learning programmes in Hong Kong. Lang. Educ. 24, 215–238. 10.1080/09500780903576125

[B56] Mackey A. Goo J. (2007). “Interaction in SLA: a meta-analysis and research synthesis,” in Conversational Interaction in Second Language Acquisition, ed A. Mackey (Oxford: Oxford University Press), 407–452.

[B57] Madini A. A. Alshaikhi D. (2017). VR for teaching ESP vocabulary: a myth or a possibility. Int. J. Engl. Lang. Educ. 5, 111–126. 10.5296/ijele.v5i2.11993

[B58] McCarthy P. M. Jarvis S. (2007) vocd: A theoretical empirical evaluation. Lang. Test. 24, 459–488. 10.1177/0265532207080767

[B59] McCarthy P. M. Jarvis S. (2010). MTLD, vocd-D, and HD-D: a validation study of sophisticated approaches to lexical diversity assessment. Behav. Res. Methods 42, 381–392. 10.3758/BRM.42.2.381 20479170

[B60] McCrocklin S. M. (2016). Pronunciation learner autonomy: the potential of automatic speech recognition. System 57, 25–42. 10.1016/j.system.2015.12.013

[B61] McCrocklin S. M. (2019). ASR-based dictation practice for second language pronunciation improvement. J. Second Lang. Pronunciation 5, 98–118. 10.1075/jslp.16034.mcc

[B62] Michel M. (2017). “Complexity, accuracy and fluency in L2 production,” in The Routledge Handbook of Instructed Second Language Acquisition, eds S. Loewen and M. Sato (New York, NY: Routledge), 50–68.

[B63] Miles J. Shevlin M. (2001). Applying Regression and Correlation: A Guide for Students and Researchers. London: Sage Publications.

[B64] Milton J. (2013). “Measuring the contribution of vocabulary knowledge to proficiency in the four skills,” in L2 Vocabulary Acquisition, Knowledge and Use: New Perspectives on Assessment and Corpus Analysis, eds C. Bardel, C. Lindqvist, and B. Laufer (EUROSLA monograph 2), 57–78.

[B65] Mroz A. (2018). Seeing how people hear you: French learners experiencing intelligibility through automatic speech recognition. Foreign Lang Ann. 51, 617–637. 10.111/flan.12348

[B66] Nation I. S. P. (2006). How large a vocabulary is needed for reading and listening? Can. Mod. Lang. Rev. 63, 59–82. 10.3138/cmlr.63.1.59

[B67] Neri A. Mich O. Gerosa M. Giuliani D. (2008). The effectiveness of computer assisted pronunciation training for foreign language learning by children. Comput. Assist. Lang. Learn. 21, 393–408. 10.1080/09588220802447651

[B68] Norris J. M. Ortega L. (2009). Towards an organic approach to investigating CAF in instructed SLA: the case of complexity. Appl. Linguist. 30, 555–578. 10.1093/applin/amp044

[B69] Pallotti G. (2009). CAF: defining, refining and differentiating constructs. Appl. Linguist. 30, 590–601. 10.1093/applin/amp045

[B70] Patanasorn C. (2010). Effects of Procedural Content and Task Repetition on Accuracy and Fluency in an EFL Contexts. PhD dissertation, Northern Arizona University, Flagstaff.

[B71] Penning de Vries B. Cucchiarini C. Bodnar S. Strik H. van Hout R. (2014). Spoken grammar practice and feedback in an ASR-based CALL system. Comput. Assist. Lang. Learn. 28, 550–576. 10.1080/09588221.2014.889713

[B72] Penning de Vries B. Cucchiarini C. Strik H. van Hout R. (2020). Spoken grammar practice in CALL: the effect of corrective feedback and education level in adult L2 learning. Lang. Teach. Res. 24, 714–735. 10.1177/1362168818819027

[B73] Polio C. Shea M. C. (2014). An investigation into current measures of linguistic accuracy in second language writing research. J. Second Lang. Writing. 26, 10–27. 10.1016/j.jslw.2014.09.003

[B74] Rahman A. A. Angraeni A. (2020). Empowering learners with role-playing game for vocabulary mastery. Int. J. Learn. Teach. Educ. Res. 19, 60–73. 10.26803/ijlter.19.1.4

[B75] Rashtchi M. Yousefi L. M. (2017). Reading input flooding versus listening input flooding: can they boost speaking skill? J. Lang. Cult. Educ. 5, 39–58. 10.1515/jolace-2017-0003

[B76] Rassaei E. (2014). Scaffolded feedback, recasts, and L2 development: a sociocultural perspective. Mod. Lang. J. 98, 417–431. 10.1111/j.1540-4781.2014.12060.x

[B77] Rassaei E. (2020). Effects of mobile-mediated dynamic and nondynamic glosses on L2 vocabulary learning: a sociocultural perspective. Mod. Lang. J. 104, 284–303. 10.1111/modl.12629

[B78] Rassaei E. (2021). Implementing mobile-mediated dynamic assessment for teaching request forms to EFL learners. Comput. Assist. Lang. Learn. 1–31. 10.1080/09588221.2021.1912105

[B79] Richards B. J. Malvern D. D. (1997). Quantifying Lexical Diversity in the Study of Language Development. Reading: University of Reading New Bulmershe Papers.

[B80] Sample E. Michel M. (2014). An exploratory study into trade-off effects of complexity, accuracy, and fluency on young learners' oral task repetition. TESL Can. J. 31:23. 10.18806/tesl.v31i0.1185

[B81] Schmitt N. (2010). Researching Vocabulary. Nottingham: Palgrave Macmillan.

[B82] Segalowitz N. (2010). The Cognitive Bases of Second Language Fluency. New York, NY: Routledge. 10.4324/9780203851357

[B83] Skehan P. (1996). “Second language acquisition and task-based instruction,” in Challenge and Change in Language Teaching, eds J. Willis, and D. Willis (Oxford: Heinemann), 17–30.

[B84] Skehan P. (1998). A Cognitive Approach to Language Learning. Oxford: Oxford University Press.

[B85] Skehan P. (2003). Task-based instruction. Lang. Teach. 36, 1–14. 10.1017/S026144480200188X

[B86] Skehan P. (2009). Modelling second language performance: integrating complexity, accuracy, fluency, and lexis. Appl. Linguist. 30, 510–532. 10.1093/applin/amp047

[B87] Skehan P. Foster P. (2001). “Cognition and tasks,” in Cognition and Second Language Instruction eds P. Robinson (Cambridge: Cambridge University Press), 183–205. 10.1017/CBO9781139524780.009

[B88] Soleimani H. Mohammaddokht F. Fathi J. (2022). Exploring the effect of assisted repeated reading on incidental vocabulary learning and vocabulary learning self-efficacy in an EFL context. Front. Psychol. 13:851812. 10.3389/fpsyg.2022.851812 35250786 PMC8894658

[B89] Soyoof A. Reynolds B. L. Shadiev R. Vazquez-Calvo B. (2022). A mixed-methods study of the incidental acquisition of foreign language vocabulary and healthcare knowledge through serious game play. Comput. Assist. Lang. Learn. 1–34. 10.1080/09588221.2021.2021242

[B90] Sun B. Révész A. (2021). The effects of task repetition on child EFL learners' oral performance. Can. J. Appl. Linguist. 24, 30–47. 10.37213/cjal.2021.31382

[B91] Sun Z. R. Xie K. (2020). How do students prepare in the pre-class setting of a flipped undergraduate math course? A latent profile analysis of learning behavior and the impact of achievement goals. Internet High. Educ. 46:100731. 10.1016/j.iheduc.2020.100731

[B92] Tai T. Y. Chen H. H. J. (2020). The impact of Google Assistant on adolescent EFL learners' willingness to communicate. Interactive Learn. Environ. 1–19. 10.1080/10494820.2020.1841801

[B93] Tai T. Y. Chen H. H. J. Todd G. (2020). The impact of a virtual reality app on adolescent EFL learners' vocabulary learning. Comput. Assist. Lang. Learn. 35, 892–917. 10.1080/09588221.2020.1752735

[B94] Tavakoli P. (2016). Fluency in monologic and dialogic task performance: challenges in defining and measuring L2 fluency. Int. Rev. Appl. Linguist. Lang. Test. 54, 133–150. 10.1515/iral-2016-9994

[B95] Tavakoli P. Campbell C. McCormack J. (2016). Development of speech fluency over a short period of time: effects of pedagogic intervention. TESOL Q. 50, 447–471. 10.1002/tesq.244

[B96] Teng F. (2019). The effects of video caption types and advance organizers on incidental L2 collocation learning. Comput. Educ. 142:103655. 10.1016/j.compedu.2019.103655

[B97] Teng F. (2020). Vocabulary learning through videos: captions, advance-organizer strategy, and their combination. Comput. Assist. Lang. Learn. 35, 518–550. 10.1080/09588221.2020.1720253. [Epub ahead of print].

[B98] Teng F. (2022). Incidental L2 vocabulary learning from viewing captioned videos: effects of learner-related factors. System 105:102736. 10.1016/j.system.2022.102736

[B99] Torlakovic E. Deugo D. (2004). Application of a CALL system in the acquisition of adverbs in English. Comput. Assist. Lang. Learn. 17, 203–235. 10.1080/0958822042000334244

[B100] Tweedie F. J. Baayen R. H. (1998). How variable may a constant be? Measures of lexical richness in perspective. Comput. Humanit. 32, 323–352. 10.1023/A:1001749303137

[B101] Van Waes L. Mariëlle L. (2015). Fluency in writing: a multidimensional perspective on writing fluency applied to L1 and L2. Comput. Composition. 38, 79–95. 10.1016/j.compcom.2015.09.012

[B102] Villamil O. S. de Guerrero M. C. M. (2006). “Socio-cultural theory: a framework for understanding the socio-cognitive dimensions of peer feedback,” in Feedback in Second Language Writing: Contexts and Issues, eds K. Hyland and F. Hyland (New York, NY: Cambridge University Press), 23–42. 10.1017/CBO9781139524742.004

[B103] Vygotsky L. S. (1978). Mind in Society: The Development of Higher Psychological Processes. Cambridge, MA: Harvard University Press.

[B104] Vygotsky L. S. (1987). “Thinking and speech,” in The Collected Works of L. S. Vygotsky: Vol. 1: Problems of General Psychology, eds R. W. Rieber, and A. S. Carton (New York, NY: Plenum), 39–285.

[B105] Wang Y. H. Young S. S. C. (2014). A study of the design and implementation of the ASR-based iCASL system with corrective feedback to facilitate English learning. Educ. Technol. Soc. 17, 219–233.

[B106] Webb S. Nation P. (2017). How Vocabulary Is Learned. Oxford: Oxford University Press.

[B107] Wertsch J. V. (1979). “The regulation of human action and the given-new organization of private speech,” in The Development of Self-Regulation Through Private Speech, ed G. Zivin (New York, NY: John Wiley and Sons), 79–98.

[B108] Wolfe-Quintero K. Inagaki S. Kim H. (1998). Second Language Development in Writing: Measures of Fluency, Accuracy, and Complexity. Honolulu, HI: University of Hawaii Press.

[B109] Xiao W. Q. Park M. (2021). Using automatic speech recognition to facilitate English pronunciation assessment and learning in an EFL context: pronunciation error diagnosis and pedagogical implications. Int. J. Comput. Assist. Lang. Learn. Teach. 11, 74–91. 10.4018/IJCALLT.2021070105

[B110] Zhai X. S. Chu X. Y. Chai C. S. Jong M. S. Y. Istenic A. Spector M.. (2021). A review of artificial intelligence in education from 2010 to 2020. Complexity 2021:8812542. 10.1155/2021/8812542

